# Food-Derived Pharmacological Modulators of the Nrf2/ARE Pathway: Their Role in the Treatment of Diseases

**DOI:** 10.3390/molecules26041016

**Published:** 2021-02-15

**Authors:** Feijie Zhao, Xinxin Ci, Xiaxia Man, Jiajia Li, Zhentong Wei, Songling Zhang

**Affiliations:** 1 Department of Obstetrics and Gynecology, The First Hospital of Jilin University, Changchun 130001, China; zhaofj18@mails.jlu.edu.cn (F.Z.); manxia@jlu.edu.cn (X.M.); lijiajia@jlu.edu.cn (J.L.); zhentongwei2009@163.com (Z.W.); 2 Institute of Translational Medicine, The First Hospital of Jilin University, Changchun 130001, China; cixinxin@jlu.edu.cn

**Keywords:** oxidative stress, Nrf2, bioactive compounds, food, diseases

## Abstract

Oxidative stress, which refers to unbalanced accumulation of reactive oxygen species (ROS) levels in cells, has been linked to acute and chronic diseases. Nuclear factor erythroid 2-related factor 2/antioxidant response element (Nrf2/ARE) pathway plays a vital role in regulating cytoprotective genes and enzymes in response to oxidative stress. Therefore, pharmacological regulation of Nrf2/ARE pathway is an effective method to treat several diseases that are mainly characterized by oxidative stress and inflammation. Natural products that counteract oxidative stress by modulating Nrf2 have contributed significantly to disease treatment. In this review, we focus on bioactive compounds derived from food that are Nrf2/ARE pathway regulators and describe the molecular mechanisms for regulating Nrf2 to exert favorable effects in experimental models of diseases.

## 1. Introduction

Oxidative stress plays an important role in the imbalance between pro-oxidant and antioxidant processes inside the cells. It is involved in different human pathologies, such as chronic obstructive pulmonary disease (COPD), Parkinson’s disease (PD), Alzheimer’s disease (AD), cancer, and diabetes [1]. In addition, extensive research during the last two decades has revealed that oxidative stress leads to acute inflammation, which in turn plays a causal role in most acute diseases, such as acute lung injury, acute kidney injury, fulminant hepatic failure, and septic shock. Therefore, the search for potential antioxidant pathways may lead to the discovery of prospective therapeutic targets for human diseases.

Among these antioxidant pathways, the nuclear factor erythroid 2-related factor 2/antioxidant response element (Nrf2/ARE) pathway is distinguished. Therefore, targeting the Nrf2 pathway is considered a prevention and treatment strategy for multiple diseases related to oxidative stress and inflammation [2]. There is abundant evidence that activation of the Nrf2 pathway reduces the inflammatory damage caused by oxidative stress and inhibits the occurrence of tumors, especially in the early stage [3]. Therefore, activators of Nrf2 provide a treatment option for inflammatory diseases such as lung injury, liver injury, kidney injury, COPD, PD, and AD. However, several studies have shown that overexpression of Nrf2 promotes cancer development and leads to chemotherapy resistance. Therefore, Nrf2 inhibitors are used to treat cancers in which Nrf2 is highly expressed.

Natural products that regulate Nrf2 have contributed significantly to drug discovery. Among these natural Nrf2 modulators, dietary Nrf2 regulators are widely accepted for their safety and ease of access. In the last few decades, many studies have shown that dietary Nrf2 regulators reduce the risk of various diseases and prevent or alleviate disease.

In this review, we highlight the protective role of Nrf2 in several acute and chronic diseases and summarize bioactive compounds in food that are regulators of Nrf2 and describe their molecular mechanisms in experimental models of acute and chronic diseases.

## 2. Nrf2/ARE Signaling Pathway

**Figure 1:**
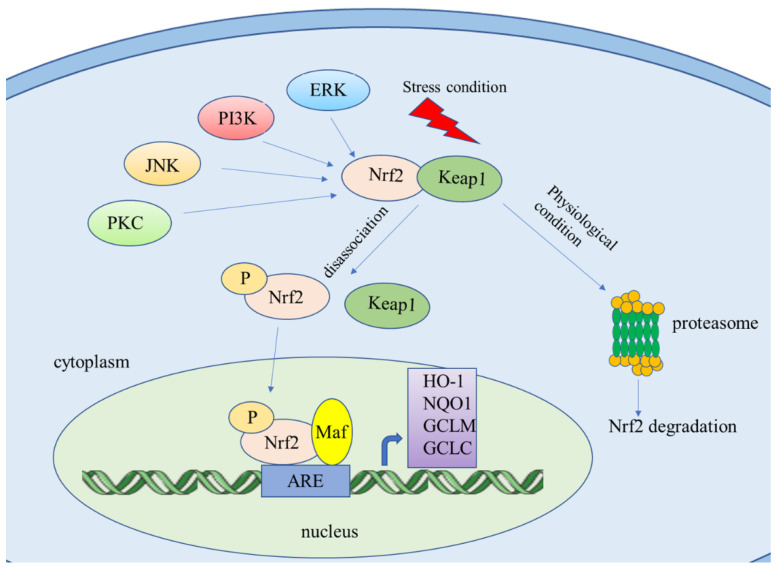
Nrf2/ARE signaling pathway. Under physiological conditions, Keap1 protein traps Nrf2 in the cytoplasm and ubiquitinates Nrf2, thereby promoting the proteolysis of Nrf2 by proteasomes. Under stress conditions, Nrf2 dissociates from the Keap1/Nrf2 complex. PKC, JNK, PIK3, and ERK can also activate Nrf2. Then, Nrf2 translocates into the nucleus, and combines with Maf and ARE to initiate transcription of downstream genes such as HO-1, NQO1, GCLM, and GCLC.

The Nrf2/ARE signaling pathway is the primary pathway for intracellular redox balance. In a normal physiological state, Nrf2 is located in the cytoplasm and binds to Keap1 through the Neh2 region [4]. It is anchored to the intracytoplasmic actin cytoskeleton in the form of the Keap1–Nrf2 complex, and it relies on E3 ubiquitin ligase to promote ubiquitin-mediated degradation of Nrf2 to maintain a basal steady-state level of Nrf2 activity [5]. Under oxidative or electrophilic stress condition, Nrf2 is released from Keap1 and translocates to the nucleus, where it forms a heterodimer with the small musculoaponeurotic fibrosarcoma (sMAF) proteins and binds to antioxidant response elements (AREs) in the promoter region of cytoprotective genes, triggering the transcription of Nrf2 target genes [5,6] (Figure 1). In addition to Keap1-dependent regulation, Nrf2 can be phosphorylated by glycogen synthase kinase-3(GSK-3) through the Neh6 domain, causing its ubiquitination by β-transducin repeat-containing protein (β-TrCP) and subsequent proteasome degradation [7].

### 2.1. Downstream Targets of Nrf2

It has been reported that Nrf2 has more than 500 target genes that are involved in redox balance, xenograft reaction, metabolism, and cell survival [3,8]. The target genes include phase I and II detoxification enzymes, growth factors, transporters, and other transcription factors [8]. The downstream genes NQO1, HO-1, and GCLC exert antioxidant effects. In addition, the downstream targets TGF-β and NF-κB are related to inflammation [9]. Furthermore, the downstream genes of Nrf2 also regulate other biological processes in human diseases, such as apoptosis, autophagy, angiogenesis, metastasis, drug resistance, differentiation, and inflammation [10]. Therefore, Nrf2 inducers exert antioxidant and anti-inflammatory effects by upregulating various cytoprotective enzymes and proteins.

### 2.2. Genes Regulate Nrf2

In addition to regulation by Keap1, the Nrf2 pathway is modulated by kinases that play a key role in phosphorylation-mediated activation of Nrf2. For example, PKC phosphorylates Nrf2 at Ser 40, resulting in the separation of Nrf2 from the Keap1–Nrf2 complex to promote the nuclear translocation of Nrf2 [8]. In addition, PI3K, JNK, ERK, and Akt also promote the activation of Nrf2 [11]. It has been reported that naringenin activates Nrf2 by both JNK and PI3K/Akt signaling [12]. Curcumin activates Nrf2 by the ERK and Akt pathways [13,14]. Moreover, resveratrol activates Nrf2 by PI3K/Akt signaling [15].

## 3. The Nrf2/ARE Pathway as a Protector in Acute Diseases

### 3.1. Acute Lung Injury

Acute lung injury is the damage of alveolar epithelial cells and capillary endothelial cells that is caused by various direct and indirect injury factors. Drugs, mechanical ventilation, and ischemia-reperfusion can all cause acute lung injury. Recent studies indicate that Nrf2 plays an important role in the antioxidant and anti-inflammatory effects of lung injury. Under hyperoxia stimulation, Nrf2-knockout mice showed greater sensitivity to lung injury with epithelium damage and inflammation than their wild-type counterparts [16]. Nrf2 shows protective effects on LPS-induced acute lung injury by inhibiting inflammation and oxidative stress [17]. In addition, in ischemia-reperfusion-induced acute lung injury, Nrf2 deficiency significantly increases the upregulation of proinflammatory factors, suggesting that Nrf2 protects against lung injury induced by ischemia-reperfusion [18].

### 3.2. Acute Kidney Injury (AKI)

Acute kidney injury (AKI) is a clinical syndrome caused by a rapid decline in renal function due to various causes. The occurrence of AKI is related to a variety of factors, including renal ischemia-reperfusion, sepsis, drugs, and other stimuli, but the specific pathogenesis is still unclear. In various kidney diseases, the level of ROS is high, and the expression of Nrf2 is downregulated [19]. In the ischemia-reperfusion-induced AKI model, compared with wild-type mice, the expression of Nrf2-regulated cell defense genes in mice that knock out Nrf2 is reduced [20]. Therefore, Nrf2 plays an important role in protecting the kidneys from damage caused by ischemia-reperfusion. In addition, cisplatin causes more severe nephrotoxicity in Nrf2-deficient mice than in wild-type mice [19]. Moreover, Nrf2 also exerts a protective effect in intravascular hemolysis-mediated AKI. AKI features, such as decreased renal function, were more severe in Nrf2-deficient mice than in wild-type mice, which exhibited downregulation of Nrf2-related antioxidant enzymes [21].

### 3.3. Acute Liver Injury

Acute liver injury refers to abnormal liver function due to various causes, including viral infection, improper drug use, food additives, excessive intake of ethanol and exposure, eating toxic food, and radiation damage. The liver can regenerate after injury, but permanent oxidative stress caused by some toxins causes liver cells fail to regenerate. Nrf2, as an important regulator of oxidative stress, plays an important regulatory role in the treatment of liver injury [22]. Compared to wild-type mice, Nrf2-knockout mice exhibit higher susceptibility to liver damage and a decreased antioxidant response to chronic ethanol consumption, drugs, etc. [8]. Low levels of serum ALT, LDH, liver hemorrhage, and necrosis were observed in Nrf2-enhanced mouse models of acute liver injury compared with those of Nrf2-null mice [23]. Moreover, in carbon tetrachloride-induced and acetaminophen-induced acute liver injury mouse models, the Nrf2 pathway plays an important role in anti-inflammatory and antioxidant effects [23]. In addition, activation of Nrf2 reduces LPS- and D-GalN-induced liver injury [24].

### 3.4. Acute Pancreatitis

Acute pancreatitis is an inflammatory response that causes self-digestion of pancreatic tissues, edema, hemorrhage, and even necrosis. Although the mechanism is not fully understood, inflammation and oxidative stress play important roles in the pathogenesis of acute pancreatitis. In an acute pancreatitis model, activation of Nrf2 decreased lipase and serum amylase levels and reduced histopathological manifestations in pancreatic tissue [25]. In Nrf2-knockout mice, pancreatic injuries substantially worsened due to exposure to acute high-dose alcohol [26]. Therefore, the regulation of Nrf2 plays an important role in the treatment of pancreatitis.

## 4. The Nrf2/ARE Pathway as a Protector in Chronic Diseases

### 4.1. PD and AD

PD and AD are neurodegenerative diseases. According to previous studies, the Nrf2/ARE pathway has become a therapeutic target for neuroprotection. PD is characterized by the loss of dopaminergic neurons in the dense substantia nigra, which causes tremors, stiffness, and bradykinesia. Studies have shown that compared to wild-type mice, Nrf2-knockout mice have a greater reduction in dopamine transporter levels in the striatum after MPTP administration [27]. Moreover, Nrf2 activation has a protective effect on dopaminergic neurons in a PD model [28]. AD is characterized by disruption of neuronal and synaptic integrity, leading to memory loss and cognitive impairment. Compared with wild-type mice, the Nrf2 deficiency AD model exhibits exacerbated neuroinflammation [29]. Moreover, Nrf2 inducers improved oxidative stress, amyloid pathology, and cognitive function in an AD mouse model [30]. Therefore, Nrf2 activators could be a potential treatment for AD and PD.

### 4.2. COPD

Chronic obstructive pulmonary disease (COPD) is a chronic respiratory disease that is characterized by continuous airflow limitation and progressive development. Many studies have shown that oxidative stress is an important inducer of COPD. Nrf2-deficient mice are more susceptible to developing more severe lung emphysema than wild-type mice, and antioxidant enzymes are suppressed [31]. Therefore, a Nrf2 inducer may be a therapy for COPD. In recent years, several natural activators of Nrf2 have been reported to have anti-inflammatory and antioxidant effects in COPD.

### 4.3. Diabetes Mellitus

Diabetes mellitus is a group of metabolic diseases characterized by chronic hyperglycemia caused by multiple etiologies and defective insulin secretion and/or utilization. Diabetes is a clinical syndrome caused by a combination of genetic and environmental factors, but its etiology and pathogenesis have not yet been fully elucidated. In Keap1-knockdown mice, Nrf2 signaling was enhanced, and activation of Nrf2 prevented the development of diabetes in type 1 diabetes mice [32]. In recent years, many bioactive ingredients in the diet that induce Nrf2 have been found to play an important role in the prevention and treatment of diabetes and its complications.

### 4.4. Cancer

Tumor refers to a local mass formed by the abnormal proliferation of cells in local tissues under the action of various tumorigenic factors. Malignant tumors can also destroy the structure and function of tissues and organs, causing necrosis, bleeding, and infection, and patients may eventually die due to organ failure. The mechanism of cancer development is intricate. It has been shown that oxidative stress plays a vital role in cancer development [33]. Oxidative stress affects all stages of the carcinogenic process, including initiation, facilitation, and progression. As a key regulator of oxidative stress, the Nrf2 pathway has become a potential target for cancer treatment [34]. Nrf2 plays a dual role in cancer. Under physiological conditions, Nrf2 maintains redox homeostasis and regulates cell growth to prevent tumorigenesis. Nrf2-deficient mice have increased susceptibility to oxidative stress and develop drug toxicity and promote carcinogenesis. Therefore, Nrf2 activation suppresses carcinogenesis in its early stage. However, continuous activation of Nrf2 promotes the development, progression, and chemotherapy resistance of cancer [3]. Continuous activation of Nrf2 not only inhibits cancer cell apoptosis, but also promotes self-renewal of cancer stem cells. Increasing evidence suggests that the inhibition of Nrf2 plays an important role in cancer treatment. One study reported that compared with cells in the control group, ovarian cells SKOV3 that were transfected with Nrf2 siRNA exhibited enhanced sensitivity to cisplatin [34]. Moreover, a growing number of diet-derived compounds, such as luteolin and diosmetin, have been shown to play an anticancer role by inhibiting Nrf2. Therefore, Nrf2 inhibition could be a way to treat cancer.

## 5. Bioactive Compounds in Food Exhibit Protective Effects in Acute Diseases

An increasing number of studies have reported that natural products play an important role in oxidative stress by regulating Nrf2. As the main ingredients in natural compounds, the bioactive substances in food are vital in the regulation of Nrf2. Dietary-derived Nrf2 regulators are mainly flavonoids, phenols, and terpenes [35]. Flavonoid Nrf2 modulators include hesperidin and quercetin. Phenolic acid Nrf2 regulators include curcumin and capsaicin. Terpene Nrf2 regulators include astaxanthin and lutein. The effects of major dietary ingredients on different acute diseases are summarized in Table 1.

### 5.1. Bioactive Compounds in Food Ameliorate Acute Lung Injury by Activating the Nrf2 Pathway

Oleanolic acid, a plant-derived triterpene, is a natural anti-inflammatory and antioxidant compound. Studies have shown that oleanolic acid improves pulmonary edema and pulmonary histological changes by upregulating the proteins SIRT1, Nrf2, and Bcl2 and downregulating the proteins NF-κB, NLRP3, and Bax [36]. It has been reported that lycium barbarum polysaccharide derived from wolfberry reduces hyperoxic acute lung injury via the activation Nrf2 mediated by AMPK [38]. In mice studies, sulforaphane from cruciferous vegetables alleviated lung injury by upregulating Nrf2 [41]. Furthermore, resveratrol, which is a polyphenol found in many plants, inhibits inflammation and oxidative stress by activating PI3K to upregulate Nrf2 expression [43].

### 5.2. Bioactive Compounds in Food Ameliorate Acute Kidney Injury by Activating the Nrf2 Pathway

Lycium barbarum polysaccharides exert antioxidant activities in AKI. In a sepsis-associated AKI rat model, lycium barbarum polysaccharides increased the expression level of Nrf2 and then decreased the expression level of NF-κB [39]. Moreover, resveratrol exerts anti-inflammatory and antioxidant effects by activating Nrf2 and its downstream target proteins in a sepsis-induced AKI model [44]. Furthermore, mangiferin, a flavonoid in mango peel, reduces renal injury by increasing Nrf2 expression and suppressing NLRP3 inflammasome activation [54]. Hesperetin is derived from citrus fruits of the rutaceae family and is one of the dihydroflavones. Our previous studies showed that hesperetin improved cisplatin-induced renal injury by activating the Nrf2 target proteins HO-1 and NQO1 and inhibiting Keap1 and Nox4 [48]. In addition, curcumin, an acidic polyphenolic usually used as a food colorant, has been shown to reduce kidney damage by regulating AMPK and the Nrf2/HO-1 pathway [52].

### 5.3. Bioactive Compounds in Food Ameliorate Acute Liver Injury by Activating the Nrf2 Pathway

Quercetin is a polyphenolic bioactive flavonoid that has been shown to have antioxidant and anti-inflammatory activities. Quercetin can treat ethanol-induced acute liver injury by inhibiting NLRP3 and activating Nrf2/HO-1 [45]. Luteolin is a kind of natural flavonoid extracted from traditional Chinese medicine. Yang et al. reported that luteolin improves HgCl-induced acute liver injury by the Sirt1/Nrf2/TNF-α signaling pathway [47]. Oxyresveratrol is a kind of natural polyhydroxystilbene rich in mulberry and has been shown to prevent liver injury by suppressing the TLR4/NF-κB pathway and activating the Keap1/Nrf2 pathway [49]. Nobiletin, an O-methylated flavonoid found in citrus peels, inhibits inflammation and oxidative stress by increasing the levels of Nrf2 and Nrf2 target proteins [50]. Oleanolic acid is a triterpene widely found in fruits, vegetables, and many medicinal materials; it was reported to protect against liver injury by activating Nrf2 and suppressing Oatp1b2 [37]. Furthermore, rosmarinic acid, one of the extracts of *Rosmarinus officinalis* Linn, upregulates Nrf2/HO-1 and downregulates the NF-κB pathway [51]. It has also been reported that curcumin protects against liver damage by regulating the Nrf2/HO-1 and TGF-β1/Smad3 pathways [53].

### 5.4. Bioactive Compounds in Food Ameliorate Acute Pancreatitis by Activating the Nrf2 Pathway

Dong et al. [40] demonstrated that lycium barbarum polysaccharide reduced inflammation by upregulating Nrf2 and HO-1 in a cerulein-induced acute pancreatitis mouse model. Moreover, sulforaphane activates Nrf2 expression and inhibits the NLRP3/ NF-κB pathway in acinar cells [42]. In addition, quercetin decreases the expression of inflammatory factors, including NF-kB, IL-1β, IL-6, and TNFα, in a rat model [46].

In conclusion, we summarized the therapeutic effects and mechanisms of natural compounds such as lycium barbarum polysaccharides, sulforaphane, and oleanolic acid on acute liver and kidney injury and other acute disease models. Although the natural compounds we describe have good therapeutic effects in some acute disease models, most of them have not yet entered clinical studies. Their pharmacodynamic properties and clinical safety need to be further studied.

## 6. Bioactive Compounds in Food in Chronic Diseases

Dietary ingredients play an important role not only in acute diseases, but also in chronic diseases. The effects of major dietary ingredients on different chronic diseases are summarized in Table 2.

### 6.1. Bioactive Compounds in Food in PD

In the MPTP-induced Parkinson’s disease model, pinostrobin plays a protective role. Pinostrobin is a natural dietary bioflavonoid found in various herbs and plants. It activates Nrf2/ARE by promoting PI3K/Akt and ERK [75]. In a 6-OHDA-induced Parkinson’s disease model, the main flavonoid in grapefruit naringenin has been shown to protect against neurotoxicity by inhibiting JNK/P38 pathway and activating the Nrf2/ARE pathway [71]. Another study showed that well-recognized Nrf2 inducer, sulforaphane, increases glutathione to exert neuroprotective effects involving mTOR, Nrf2, and autophagy [55].

### 6.2. Bioactive Compounds in Food in AD

Quercetin exerts a neuroprotective effect by regulating the JNK, MAPK, and PI3K/Akt pathways [68]. Resveratrol increases the antioxidant capacity by the Nrf2/HO-1 pathway and enhances estrogen levels in an AD model [61]. Ginsenosides are triterpenoid saponins contained in ginseng. Liu et al. [73] showed that ginsenoside Re activates Nrf2-mediated antioxidant effects and suppresses ROS/ASK-1-dependent mitochondrial apoptosis in Aβ-induced SH-SY5Y cells. In a cellular model of AD, sulforaphane decreased the inflammatory cytokines IL-1β, IL-6, and NF-κB and promoted the nuclear translocation of Nrf2 by decreasing the DNA demethylation levels of the Nrf2 promoter [56]. Other studies have also reported that the Nrf2 activator lycopene, which is a lipid carotenoid extracted from fruits and vegetables, also has a neuroprotective effect in an AD model [77]. Moreover, ellagic acid, a natural polyphenol, was reported to have a neuroprotective effect via the NF-κB/Nrf2/TLR4 pathway [76]. These results suggest that Nrf2 inducers may be potential treatment strategies for AD.

### 6.3. Bioactive Compounds in Food in COPD

Astaxanthin is a carotenoid, widely distributed in marine life such as shrimp and crab. It has been reported to improve smoking-induced emphysema and inhibit oxidative stress by activating Nrf2 and HO-1, providing a new treatment option for COPD [79]. Moreover, sulforaphane can protect lung epithelial cells by decreasing ROS levels and upregulating the expression of Nrf2 [57,58]. Similarly, resveratrol also exerts an antioxidative effect by activating Nrf2 [62]. Furthermore, quercetin restores corticosteroid sensitivity of COPD by activating Nrf2 through activating AMPK [69]. These studies show that dietary Nrf2 activators may be a way to prevent and treat COPD.

### 6.4. Bioactive Compounds in Food in Diabetes Mellitus

In a rat model of diabetes, mangiferin has been shown to promote wound healing caused by diabetes by upregulating the expression of Nrf2, VEGF, and PI3K and downregulating the expression of TNFα and NF-κB p65 [65]. Studies show that resveratrol reduces myocardial oxidative stress and ischemia-reperfusion injury caused by diabetes by activating Nrf2 expression through stimulation of SIRT1 or inhibition of GSK3β [63]. Moreover, lutein increases Nrf2 nuclear translocation by activating ERK and Akt, thereby protecting retinal pigment epithelium from diabetes-related damage [80]. In a streptozotocin-induced type 1 mouse diabetes model, ginsenoside Rg1 promotes insulin secretion and reduces blood glucose. In addition, protopanaxtriol type saponin ginsenoside Rg1 decreases the inflammatory factors IL-1β and IL-18 in the blood and reduces inflammatory effects in the liver and pancreas through the NLRP3 and Keap1/Nrf2/HO-1 pathways [72]. Moreover, sulforaphane plays a role in preventing the progression of type 2 diabetes-induced cardiomyopathy. Mechanistically, sulforaphane increases the expression level of Nrf2 via regulation of the AMPK/Akt/GSK3β pathway [59]. Furthermore, hesperetin also plays a protective role in diabetic nephropathy via activation of the Nrf2/ARE pathway [67].

In summary, we have described the therapeutic effects of some dietary natural compounds, including naringenin, quercetin, and sulforaphane, on AD, PD, COPD, and diabetes. Although some natural compounds have entered clinical studies, most of them are still limited to laboratory studies due to their safety, and detailed mechanisms of their effects are not completely clear. Therefore, more in-depth research is needed to provide the basis for its clinical application.

### 6.5. Bioactive Compounds in Food in Cancer

#### 6.5.1. Lung Cancer

Lung cancer is the most common primary lung tumor with one of the fastest growing morbidity and mortality rates and is one of the most dangerous diseases. Cigarette smoke has been established as the primary etiological factor for the disease.

A previous study showed that ginsenoside Rd, a main active component of ginsenosides, inhibits the proliferation of A549 cells and A549/DDP cells. Ginsenoside Rd reverses cisplatin resistance in A549/DDP cells by inhibiting the expression of Nrf2 and Nrf2 target proteins [88]. Similarly, luteolin enhances the chemotherapy sensitivity of A549 cells by promoting the degradation of Nrf2 mRNA and reducing Nrf2 binding to AREs [74]. Retinoic acid is a transformed form of vitamin A in cells. Retinoic acid combined with cisplatin has a beneficial effect in the treatment of lung cancer by inhibiting Nrf2 and Nrf2 target genes. In addition, combination therapy also increased autophagy and had a beneficial effect in clinical trials [83]. Moreover, in vitro and in vivo, diosmetin, which is a natural flavonoid found in beans and citrus plants, improves the chemotherapeutic sensitivity of lung cancer to paclitaxel by inhibiting Nrf2 [84]. In addition, curcumin suppresses the migration and invasion of lung cancer cells by inhibiting the PI3K/Akt/mTOR pathway [86].

#### 6.5.2. Liver Cancer

Liver cancer is one of the most common types of cancer, ranking fifth in the world. The etiology and exact molecular mechanism of liver cancer is not fully understood, and its pathogenesis is currently considered to be a multifactor and multistep complex process. Oxidative stress is an important factor in the development and progression of liver cancer [89].

In thioacetamide (TAA)-induced hepatocellular carcinoma, vitamin D increases the antitumor effect of 5-FU by modulating the expression levels of TGF-β1, caspase-3, and Nrf2 [66]. Apigenin, a natural bioflavonoid, which is widely found in many fruits and vegetables, increases doxorubicin chemotherapy sensitivity in both BEL-7402 cells and BEL-7402 xenografts by decreasing Nrf2 expression at both the RNA and protein levels and decreasing Nrf2 target proteins through the PI3K/Akt pathway [85].

#### 6.5.3. Ovarian Cancer

Ovarian cancer is a common female reproductive system tumor, with the third highest incidence of gynecological malignancies, but its mortality rate ranks first among gynecological malignancies [90]. Ovarian cancer is prone to relapse and drug resistance, and the prognosis is poor. In recent years, many studies have shown that oxidative stress is involved in the pathogenesis of ovarian cancer [91]. Many chemotherapy drugs inhibit proliferation and metastasis and induce apoptosis of ovarian cancer cells by regulating oxidative stress.

Studies have found that a high dose of ascorbate acts as a pro-oxidant to increase chemosensitivity in ovarian cancer by inducing DNA damage and regulating the AMPK/MTOR pathway [81]. Other research shows that tangeretin, a citrus flavonoid, reverses cisplatin resistance in ovarian cancer and enhances cisplatin sensitivity of ovarian cancer by inhibiting the PI3K/Akt signaling pathway [82]. Other studies also report that lycopene exerts anticancer effects in ovarian cancer by inhibiting ERK signaling [78].

#### 6.5.4. Breast Cancer

Breast cancer is a malignant tumor that occurs in epithelial tissue of the breast glands. Breast cancer is not only one of the most common malignant tumors in women worldwide, but also the leading cause of cancer death in women [92]. Increasing experimental and clinical evidence indicates that reactive oxygen species and their reactive derivatives are involved in the occurrence and development of breast cancer [93]. In recent years, several natural compounds found in the diet have been shown to prevent and treat breast cancer by regulating oxidative stress.

Nobiletin was verified to inhibit proliferation and migration and promote apoptosis in MCF-7 breast cancer cells. Nobiletin inhibits Nrf2 and NF-κB translocation into the nucleus and activates the phosphorylation of P38 [70]. In addition, curcumin promotes the nuclear translocation of Nrf2 and decreases the expression of Fen1 to exert anticancer effects [87]. However, other studies also report that the activation Nrf2 can prevent occurrence of cancer. Sulforaphane and resveratrol upregulate Nrf2 in breast epithelial cells to reduce the risk of breast cancer [60,64].

Nrf2 is a double-edged sword in cancer. The activation of Nrf2 can prevent the occurrence of cancer to a certain extent, but the continuous activation of Nrf2 can lead to cancer progression and drug resistance. Therefore, inhibition of Nrf2 is a potential strategy for cancer treatment. In this part, we have summarized some natural compounds as Nrf2 inhibitors to play a therapeutic role in cancer. However, some studies have also shown that some compounds may trigger the activation of Nrf2 while exerting anti-cancer effects. Therefore, their value as Nrf2 inhibitors is very controversial.

## 7. Clinical Application of Food-Derived Nrf2 Modulator

Although many natural compounds have been reported to have definite therapeutic effects in cells and animals, most of them have unknown effects in humans. Several Nrf2 modulators are currently in clinical use or in clinical trials. Among them, sulforaphane has been studied extensively, and sulforaphane has been successfully used to treat patients with type II diabetes [94]. A clinical trial has shown that curcumin can lower blood sugar in people with type 2 diabetes [95]. Resveratrol is also in phase III clinical trials in chronic renal insufficiency and Huntington disease. Results from a phase I clinical trial showed that quercetin exhibited antiviral activity in patients with hepatitis C [96]. In addition, some diet-derived compounds such as genistein and astaxanthin have also entered clinical trials at different stages of different diseases [97,98].

## 8. Conclusions

Oxidative stress is involved in the occurrence and development of many diseases, including liver and lung damage, diabetes, neurodegenerative diseases, and cancer. Nrf2 is a major antioxidant that plays an important role in antioxidant and anti-inflammatory activities. Keap1/Nrf2 is a vital signaling pathway for the treatment of diseases that are characterized by oxidation and inflammation. Therefore, targeting Nrf2 is emerging as a treatment strategy for inflammation and oxidative stress-related acute and chronic diseases such as acute lung injury, AKI, cancer, and diabetes.

In recent years, an increasing number of Nrf2 regulators have been discovered, among which bioactive compounds in food have attracted much attention. These compounds can play an antioxidant, anti-inflammatory, and anti-tumor role in the prevention and treatment of disease. Most Nrf2 activators are electrophilic molecules that modify cysteine residues in thiol-rich Keap1 proteins by oxidation or alkylation covalently. The relative titers activated by different types of inducers are related to their ability to release electrons [99]. It is important to chemically modify electrophiles according to their sulfhydryl reactivity to optimize their therapeutic potential [100]. However, most of the known Nrf2 regulators have low bioavailability and lack specificity, and their safety is not clear, which limits their clinical promotion.

In general, many natural compounds have significant effects in cell and animal experiments. However, their clinical application is full of challenges. Further studies are needed to verify their clinical efficacy.

## Figures and Tables

**Table 1:** The effects of bioactive compounds of food on acute diseases.

Compound	Disease	Effect	Ref.
**Oleanolic acid**	acute lung injury	improves pulmonary edema and pulmonary histological changes	[36]
	acute liver injury	protects against phalloidin-induced liver injury	[37]
**Lycium barbarum polysaccharide**	acute lung injury	reduces hyperoxic acute lung injury	[38]
	AKI	protects sepsis-induced AKI	[39]
	acute pancreatitis	reduces inflammation in cerulein induced acute pancreatitis	[40]
**Sulforaphane**	acute lung injury	anti-inflammation and anti-oxidation	[41]
	acute pancreatitis	protects pancreatic acinar cell injury	[42]
**Resveratrol**	acute lung injury	protects sepsis-induced lung injury	[43]
	AKI	ameliorates sepsis-induced AKI in a pediatric rat model	[44]
**Quercetin**	acute liver injury	inhibits NLRP3 and activates Nrf2/HO-1	[45]
	acute pancreatitis	decreases the expression of inflammatory factors	[46]
**Luteolin**	acute liver injury	improves HgCl-induced liver injury	[47]
**Mangiferin**	AKI	attenuates sepsis-induced AKI	[48]
**Hesperetin**	AKI	reduces oxidative stress, inflammation and apoptosis	[48]
**Oxyresveratrol**	acute liver injury	prevents LPS/D-galactosamine-induced acute liver injury in mice	[49]
**Nobiletin**	acute liver injury	attenuates LPS/D-galactosamine-induced liver injury in mice	[50]
**Rosmarinic acid**	acute liver injury	prevents LPS/D-galactosamine-induced acute liver injury in mice	[51]
**Curcumin**	AKI	reduces kidney damage	[52]
	acute liver injury	protects liver damage by regulating Nrf2/HO-1	[53]

**Table 2:** The effects of bioactive compounds of food on chronic diseases.

Compound	Diseases	Effect	Ref.
**Sulforaphane**	PD	increases glutathione to play neuroprotective effects	[55]
	AD	decreases inflammatory cytokines level	[56]
	COPD	protects lung epithelial cells by decreasing ROS level	[57,58]
	diabetes	prevents the progression of type 2 diabetes-induced cardiomyopathy	[59]
	breast cancer	prevents the occurrence of breast cancer	[60]
**Resveratrol**	AD	increases antioxidant capacity by Nrf2/HO-1 pathway	[61]
	COPD	anti-inflammation and anti-oxidation	[62]
	diabetes	reduces myocardial ischemia-reperfusion injury caused by diabetes	[63]
	breast cancer	prevents the occurrence of breast cancer	[64]
**Mangiferin**	diabetes	promotes wound healing caused by diabetes	[65]
**vitamin D**	liver cancer	increases the antitumor effect of 5-FU	[66]
**Hesperetin**	diabetes	ameliorates diabetic nephropathy in rats	[67]
**Quercetin**	AD	regulates JNK, MAPK, and PI3K/Akt pathways	[68]
	COPD	prevent lung disease progression	[69]
**Nobiletin**	breast cancer	inhibits proliferation, migration, and promotes apoptosis	[70]
**Naringenin**	PD	protects against 6-OHDA-induced neurotoxicity	[71]
**Ginsenoside Rg1**	diabetes	promotes insulin secretion and reduces blood glucose	[72]
**Ginsenoside Re**	AD	activate Nrf2 and suppress ROS/ASK-1 dependent mitochondrial apoptosis	[73]
**Ginsenoside Rd**	lung cancer	inhibits proliferation and reverse cisplatin resistance	[74]
**Pinostrobin**	PD	has neuroprotection in neurotoxin-induced PD through activating Nrf2/ARE signaling	[75]
**Ellagic acid**	AD	has neuroprotective effect by NF-κB/Nrf2/TLR4 pathway	[76]
**Lycopene**	AD	inhibits the inflammatory response caused by oxidative stress	[77]
	ovarian cancer	inhibits proliferation and induces apoptosis	[78]
**Astaxanthin**	COPD	improves smoking-induced emphysema	[79]
**Lutein**	diabetes	protect retinal pigment epithelium from diabetes-associated damage	[80]
**Ascorbate**	ovarian cancer	increases chemosensitivity	[81]
**Luteolin**	lung cancer	enhances chemotherapy sensitivity of A549 cell	[74]
**Tangeretin**	ovarian cancer	reverses the cisplatin resistance	[82]
**Retinoic acid**	lung cancer	promotes A549 cells sensitization to cisplatin	[83]
**Diosmetin**	lung cancer	increases chemotherapy sensitivity of lung cancer to paclitaxel	[84]
**Apigenin**	liver cancer	increases doxorubicin chemotherapy sensitivity	[85]
**Curcumin**	lung cancer	anti-lung cancer through multiple mechanisms	[86]
	breast cancer	promotes nuclear translocation of Nrf2	[87]

## Data Availability

Data is contained within the article.

## Sample Availability

Samples of the compounds are available from the authors.
